# CMR to stratify post-TAVR paravalvular leak in patients with suboptimal echocardiography

**DOI:** 10.1186/1532-429X-16-S1-O102

**Published:** 2014-01-16

**Authors:** Gregory Hartlage, Salim Hayek, Vasilis Babaliaros, Patricia Keegan, Vinod Thourani, Stamatios Lerakis

**Affiliations:** 1 Structural Heart and Valve Center, Division of Cardiology, Emory University School of Medicine, Atlanta, Georgia, USA

## Background

Despite extensive pre-procedure evaluation, greater than mild paravalvular leak (PVL) occurs in over 10% of patients undergoing transcatheter aortic valve replacement (TAVR) and is associated with worse outcomes. Echocardiography, the standard method of imaging PVL, often has limited utility due to frequently multiple eccentric regurgitant jets. Acoustic shadowing from the valve stent and native aortic valve calcification may lead to further underestimation of PVL. Cardiovascular magnetic resonance (CMR) is considered the gold standard for quantification of valvular regurgitation. We evaluated the utility of CMR to grade PVL severity and predict outcomes in patients with suboptimal echocardiography.

## Methods

Seventeen non-operative post-TAVR patients (NYHA class III-IV; age 84 ± 5 yrs) underwent CMR due to PVL and symptoms out of proportion to echocardiographic findings or suboptimal echocardiographic study. CMR was performed on a Siemens Avanto 1.5 T with velocity phase imaging in the ascending aorta for flow quantification. CMR PVL severity was graded by regurgitant fraction (RF; mild≤20%, moderate 21-39%, severe ≥40%). Short- and intermediate-term follow-up was conducted after CMR. Patients were followed-up for symptoms and a composite outcome of repeat invasive therapy, heart failure hospitalization, or death.

## Results

CMR was performed as early as 2 days and as late as 35 months post-TAVR in both the inpatient (n = 9) and outpatient (n = 8) settings. PVL grading was mild in 11, moderate in 3, and severe in 3 patients. Compared to echocardiography, PVL severity was reclassified in 47% (5 upgraded to moderate or severe; 3 downgraded to mild). CMR findings guided further management in 71% (medical therapy including diuresis in 8 with mild or moderate PVL; invasive therapy including vascular plug closure or valve in valve deployment in 4 with moderate or severe PVL). At short-term follow-up (mean 1.7 ± 1.4 months), 76% had NYHA class improvement. At intermediate-term follow-up (mean 11.6 ± 1.0 months), 65% had persistent improvement in NYHA class (Table 1). At one year, 47% (n = 8) met the composite outcome. Outcome free survival was significantly better with ≤mild PVL compared to > mild PVL (73% vs 17%; p < 0.05). The mean RF was larger in those reaching the composite outcome compared to those not (24 ± 19% vs 11 ± 8%), with a trend towards significance (p = 0.08; Table 2). Patients undergoing repeat CMR after invasive PVL therapy (n = 3) demonstrated a reduction in mean RF from 43% to 12%.

**Table 1:** CMR clinical utility and follow-up symptoms.

	Category	Result
**CMR clinical utility**	PVL grading reclassification	47% (18/17)
	Guided further management	71% (12/17)
**Follow-up symptoms**	Short-term (≤3 months) NYHA class improvement to I or II	76% (13/17)
	Intermediate-term (~1 year) Persistent NYHA class improvement	65% (11/17)

CMR = cardiovascular magnetic resonance; PVL = paravalvular leak; NYHA = New York Heart Association.

**Table 2:** CMR PVL grading and prognosis.

	Category	Result	Significance
**Prognosis**	≤Mild PVL	73% outcome* free survival	p < 0.05
	> Mild PVL	17% outcome* free survival	
	- Composite outcome*	Mean RF 11 ± 8%	p = 0.08
	+ Composite outcome*	Mean RF 24 ± 19%	

PVL = paravalvular leak; RF = regurgitant fraction; *Composite outcome = repeat invasive therapy, heart failure hospitalization, or death within a year of CMR.

## Conclusions

CMR stratifies PVL severity in symptomatic patients with suboptimal echocardiography or discrepant results. CMR is useful in guidance of specific post procedure therapy and has prognostic importance, with greater than mild PVL (RF > 20%) associated significantly with worse outcomes.

## Funding

None.

